# Serum lipidomic and metabolomic signatures link epicardial adipose tissue to cardiovascular diseases in SLE: a post-hoc analysis

**DOI:** 10.1136/lupus-2026-001993

**Published:** 2026-04-21

**Authors:** Levi-dan Azoulay, Fanny Urbain, Maharajah Ponnaiah, Farid Ichou, Marie Lhomme, Nadjia Kachenoura, Lyza Ben Bouazza, Anatol Kontush, Philippe Lesnik, Maryse Guerin, Stephane Hatem, Micheline Pha, Miguel Hie, Alexis Mathian, Matthias Papo, Fleur Cohen-Aubart, Julien Haroche, Alban Redheuil, Zahir Amoura, Wilfried Le Goff

**Affiliations:** 1 Service de Médecine Interne 2, Sorbonne Université, Assistance Publique–Hôpitaux de Paris (APHP), Groupement Hospitalier Pitié-Salpêtrière (GHPS), Centre de Référence des maladies auto-immunes et auto-inflammatoires systémiques rares de l'adulte d'Ile-de-France, Centre et Martinique, Institut E3M, Inserm UMRS, Centre d’Immunologie et des Maladies Infectieuses (CIMI-Paris), Paris, France; 2 Laboratoire d'Imagerie Biomédicale, Sorbonne Université, INSERM, CNRS, Paris, France; 3 Service de Médecine Interne et d’Immunologie Clinique, Université Paris-Saclay, Gif-sur-Yvette, France; 4 Foundation for Innovation in Cardiometabolism and Nutrition, ICAN, Paris, France; 5 Unité de Recherche sur les Maladies Cardiovasculaires et Métaboliques, Sorbonne Université, Paris, France; 6 Imagerie Cardio-Thoracique, Sorbonne Université, Assistance Publique-Hôpitaux de Paris, Institut de Cardiologie, Hôpital Pitié-Salpêtrière, Paris, France

**Keywords:** Systemic Lupus Erythematosus, Cardiovascular Diseases, Atherosclerosis, Autoimmunity

## Abstract

**Objective:**

Cardiovascular diseases are a leading cause of morbidity and mortality in SLE. In this target population, epicardial adipose tissue (EAT) volume is increased, but its determinants and roles are poorly understood. This study aimed to assess the circulating metabolomic and lipidomic signatures associated with EAT in SLE.

**Methods:**

A comprehensive analysis of the circulating metabolome and lipidome was conducted in a monocentric and retrospective cohort of patients with SLE followed at the French Referral Center (Pitié-Salpêtrière Hospital) who underwent a cardiac CT scan. Coronary artery calcium (CAC) scores and EAT volumes were collected. An increased indexed EAT (EATi) volume was defined by an EAT volume >68 mL/m^2^ as previously described.

**Results:**

A total of 179 SLE patients were included (mean age, 43±14 years; 100% female). Mean EATi volume was 63±37 mL/m^2^ and was increased (>68 mL/m^2^) in 61 patients (34.1%). Increased EATi volume was significantly associated with age, body mass index, triglyceride levels, CAC score, prior atherosclerotic cardiovascular disease and 47 metabolites/lipid species. A stepwise linear regression identified five key metabolites and lipid species independently associated with EATi volume. A lipidomic and metabolic signature yielded a good area under the curve for EATi volume status prediction (0.80; 95% CI 0.73 to 0.87).

**Conclusions:**

EAT volume is increased and associated with a distinct serum lipidomic and metabolomic signature in SLE.

**WHAT IS ALREADY KNOWN ON THIS TOPIC**

- SLE patients have an increased cardiovascular risk that is not fully explained by traditional factors. Epicardial adipose tissue (EAT) is increased in SLE and associated with coronary atherosclerosis, but the mechanisms underlying its expansion, particularly its metabolic and lipidomic determinants, remain unknown, warranting further investigation.

**WHAT THIS STUDY ADDS**

- This study identifies a specific circulating lipidomic and metabolomic signature associated with EAT volume in SLE. These findings highlight novel metabolic pathways, including very-long chain lipids and microbiota-related metabolites, that may contribute to EAT expansion and increased cardiovascular risk in SLE.

**HOW THIS STUDY MIGHT AFFECT RESEARCH, PRACTICE OR POLICY**

- This study suggests that CT-derived epicardial adipose tissue volume assessment and circulating lipidomic and metabolomic profiling could have value in improving cardiovascular risk stratification in patients with SLE beyond traditional tools. By identifying cardiometabolic pathways and biomarkers linking epicardial adipose tissue to coronary atherosclerosis, this study supports a metabolic-driven approach to cardiovascular risk stratification in SLE and highlights EAT as a potential therapeutic target, paving the way for novel strategies including sodium-glucose cotransporter 2 inhibitors (SGLT2i) and glucagon-like peptide-1 receptor agonists (GLP-1 RA).

## Introduction

 SLE patients are at increased risk for atherosclerosis and cardiovascular events.^1^,^3^ Yet, predicting and explaining this increased risk remain challenging.^4^ Among the currently available tools, epicardial adipose tissue (EAT) volume is a promising imaging biomarker that is significantly associated with incident cardiovascular events in the general population.^5^ Prior works have established that EAT volume was increased in SLE patients and associated with coronary artery calcium (CAC), a robust predictor of coronary heart disease.^6^ However, no studies have further explored the pathophysiology of EAT increased volume in SLE. Yet, understanding the mechanisms underlying the development of EAT could help guide future therapeutic approaches.^7^

In the past decade, omics-based technologies have enabled the understanding of a broad range of biological mechanisms at stake in various diseases.^8^ Prior analyses of the lipidome in EAT revealed a specific lipid signature associated with coronary artery disease (CAD) which was in part reflected at the plasma level.^9^ Further, our team previously identified circulating metabolomic and lipidomic biomarkers as predictors of cardiovascular risk in SLE. But whether EAT could mediate this risk is unknown.^10^

The main aim of this study was to assess the circulating metabolomic and lipidomic signatures associated with EAT in SLE.

## Methods

### Study population

Patients with SLE followed at the French National Reference Center for SLE (Pitié-Salpêtrière Hospital, Paris, France) who underwent both a non-contrast cardiac CT imaging and a metabolomic and lipidomic sample analysis between January 2014 and December 2017 were retrospectively included. SLE was defined according to the American College of Rheumatology and European Alliance of Associations of Rheumatology 2019 criteria. Imaging was performed in routine care as part of a cardiovascular risk assessment in a day care hospital setting.

CT-based cardiovascular assessments were performed at the discretion of the physician in routine practice irrespective of their clinical presentation in accordance with the current policy of the French Referral Center for SLE. Such policy is supported by pivotal data published in the early 2000s and by current guidelines and robust data suggesting that patients with autoimmune diseases bear a higher cardiovascular risk that is poorly captured by contemporary risk scores.^14 11^,^14^

The present study is a post-hoc analysis of a patient cohort whose data were described in our previous publication.^10^ In this study, we identified metabolic and lipidomic alterations associated with the presence of CAC in patients with SLE, including disruptions in purine, arginine and proline metabolism, gut-derived metabolites and elevated circulating ceramides with very long-chain fatty acids. Of the 211 patients included in the original study, 32 patients were excluded owing to absence of on-site CT data because it precluded extracting EAT volumes in these patients.

### Baseline clinical assessment

Duration of the disease, clinical involvements (skin, serositis, haematology, joints and nervous system), treatment regimens (steroids, methotrexate and hydroxychloroquine) and SLE variables (C3 complement component, anti-double-stranded DNA (dsDNA), ANA and antiphospholipid (aPL) antibodies) were collected. Disease activity was estimated by the Systemic Lupus Erythematosus Disease Activity Index (SLEDAI) score. Atherosclerotic cardiovascular disease (ASCVD) was defined by prior acute coronary syndrome, ischaemic stroke or transient ischaemic attack and symptomatic peripheral artery disease. The presence of cardiovascular risk factors, including hypertension, hypercholesterolaemia, diabetes, obesity, smoking status, family or personal history of cardiovascular events and inflammation (ultrasensitive C-reactive protein (US-CRP)) was determined for all patients. Intima-media thickness was manually measured on the common carotid artery during routine ultrasonography performed by experienced radiologists from our functional diagnostics laboratory (Internal Medicine, Metabolism and Endocrinology Institute, Pitié-Salpêtrière Hospital).

### CT imaging acquisition and analysis

All patients underwent a non-contrast cardiac-gated CT at baseline. CT exams were performed on either a dual source SOMATOM Definition Flash or EDGE scanner (Siemens Healthineers, Erlangen, Germany). Imaging was performed from the base of the aortic arch to the apex of the heart using the following scan parameters: 120 kVp and mAs varying with patient body size. Imaging was prospectively triggered in mid-diastole and 3-mm-thick slices were used for CAC and EAT analysis.

The EAT volume in mL was measured through an automated artificial intelligence (AI)-based segmentation of the cardiac area with a thresholding centred on the density range of adipose tissue values (−190 to −30 Hounsfield units) using *syngo*.via Frontier, Cardiac Risk Assessment software (Siemens Healthineers, Germany). EAT volumes were divided by body surface area (obtained using the Mosteller formula^15^) resulting in an indexed EAT (EATi) in mL/m^2^. EATi was further dichotomised according to a previously validated threshold (≤ or >68 mL/m^2^).^16^ CAC scoring was performed using the *syngo.*via CT CAC scoring software (Siemens Healthineers, Germany) according to the standard Agatston scoring method using measured calcified coronary plaque area (mm^2^) and peak calcium density on a per lesion basis over the entire epicardial coronary tree.^17^

Image analyses were performed using automated AI validated tools from *syngo.via* (Siemens Healthineers, Germany) that are currently validated for clinical use (CAC score) or have been previously employed in published studies (EAT volume).^18^,^21^ Regarding AI-derived EAT volume, the method leverages Hounsfield units, providing intrinsic robustness, and all results were additionally checked through visual verification by a single reader (L-dA) to ensure accuracy and reliability.

### Metabolomic and lipidomic profiling

Serum lipids and metabolites were quantified by Liquid Chromatography – Electrospray Ionization – Tandem Mass Spectrometry (LC-MS/MS) and Liquid Chromatography - High-Resolution Mass Spectrometry (LC-HRMS), respectively, as previously reported.^10^ Omics data are available in Metabolomics Workbench (ID#ST002732, datatrackID:4069) and MetaboLights (ID#MTBLS7031).

### Statistical analysis

The statistical analysis of the lipidomics and metabolomics data was performed using Multi Experiment Viewer statistical software package (V.4.9.0). Affected metabolic pathways were identified using the MetExplore V.2.30.10 web-based tool. The metabolic network (source database: KEGG Map) of human species was based on 90 metabolic pathways including 1618 metabolites, 2067 reactions, 1456 enzymatic complexes and 1456 genes. Metabolic networks are directed graphs, so it is possible to calculate compound importance based on relative betweenness centrality and out-degree centrality of any given compound from a pathway.

Statistical analyses were performed in four steps using R software, V.4.2.3 (R Project for Statistical Computing).

First, a description of the study population was performed. Normally distributed continuous variables are expressed as a mean±SD, and non-normally distributed continuous variables are reported as median with IQR. Categorical variables are expressed as frequencies and percentages.

Second, clinical and biological characteristics of patients according to the EATi volume status were compared, using a cut-off point of 68 mL/m^2^, as previously reported.^16^ Comparisons were performed with χ^2^ or Fisher’s exact tests for categorical variables and with Student’s t-tests or Mann-Whitney-Wilcoxon tests for continuous variables. Then, a multivariable linear regression analysis was performed to identify independent clinical and biological predictor variables associated with EATi volume. Variables were included in the model provided their p value was ≤0.1 on univariate analysis and the provided prior literature supported a potential relationship with EAT.^6^ No matching was performed; however, the multivariable regression model was used to adjust for potential confounding factors, including age, body mass index (BMI), hypertension and lipid profile.

Third, lipid species and metabolites were compared between patients with vs without increased EATi volume (as defined according to the same threshold of 68 mL/m^2^) using Student’s t-tests. Lipid species and metabolites were plotted using a heatmap. Then, lipid species and metabolites independently associated with EATi volume were identified through a lasso regression followed by a stepwise analysis. Lasso regression allowed for feature selection using the non-zero coefficient method. Variables selected from the lasso regression were included in the stepwise regression. Stepwise analysis (or sequential replacement) is a combination of forward and backward selections that starts with no predictors and then sequentially adds the most contributive predictors (forward selection). After adding each new variable, variables that no longer provide an improvement in the model fit are removed (backward selection).

Fourth, receiver operating characteristic curve (ROC) analyses were performed to assess the value of metabolites to predict EAT volume status. A principal component analysis (PCA) was performed to reduce the dimension of the lipid species and metabolites that were found to be significantly associated with EATi volume status. The first dimension of the PCA was kept as the omic signature quantitative variable. Candidate predictors included for ROC analyses were the omic (lipidomic and metabolic) signature (as encoded by the first dimension of the PCA), BMI and triglycerides (TGs).

## Results

### Study population

A total of 179 SLE patients from our cohort^10^ were included in this study (mean age, 43±14 years; 100% female). Mean SLE duration was 11 years at inclusion. Median SLEDAI was 2 (0–4). Prior organ involvement included skin manifestations in 122 patients (68%), pleural or pericardial effusions in 20 patients (11%) and lupus nephritis in 55 patients (31%). aPL antibodies were present in 52 patients (29%). A total of 158 patients (88%) were on hydroxychloroquine and 101 (56%) were on corticosteroids. A total of 11 patients (6%) had prior ASCVD. Cardiovascular risk factors were as follows: hypertension 27%, diabetes 2%, dyslipidaemia 6% and the mean BMI was 24±6 kg/m^2^. Patients’ characteristics are reported in table 1.

**Table 1:** Baseline characteristics in all patients and according to epicardial adipose tissue volume

	All patients	SLE patients with EATi ≤68 mL/m^2^	SLE patients with EATi >68 mL/m^2^	P value
Number of patients	179	118	61	–
Age at inclusion	43±14	39±12	52±13	**<0.001**
Female sex	179 (100)	118 (100)	61 (100)	–
Cardiovascular assessment				
Prior ASCVD	11 (6.1)	4 (3.3)	7 (11.5)	**0.047**
Hypertension	48 (26.8)	19 (16.1)	29 (47.5)	**<0.001**
Diabetes mellitus	4 (2.2)	2 (1.69)	2 (3.28)	0.283
Body mass index	24±6	23±4	27±7	**<0.001**
Current smokers	36 (20.1)	22 (18.6)	14 (23.0)	0.628
IMT (right), mm	0.54±0.15	0.51±0.15	0.58±0.16	**0.032**
IMT (left), mm	0.57±0.15	0.54±0.14	0.62±0.16	**0.012**
Carotid plaques	30 (18.5)	12 (11.2)	18 (32.7)	**0.002**
Chronic kidney disease	12 (6.7)	6 (5.1)	6 (9.8)	0.237
Serum creatinine, µmol/L	73±41	71±30	80±57	0.363
TG, g/L	0.9±0.4	0.8±0.4	1±0.5	**0.007**
TC, g/L	1.8±0.5	1.7±0.6	2±0.4	**0.002**
LDL-C, g/L	1.0±0.4	1±0.3	1.2±0.4	**<0.001**
HDL-C, g/L	0.6±0.2	0.6±0.2	0.6±0.2	0.498
C reactive protein	2.3±5.1	1.6±3.4	3.5±7.3	**0.01**
Lipid-lowering therapies	12 (6.7)	2 (1.7)	10 (16.4)	**0.004**
SLE features				
Cutaneous inv.	122 (68)	79 (100)	43 (97.7)	0.358
Articular inv.	156 (87)	103 (87)	53 (87)	1
Haematological inv.	46 (98)	32 (100)	14 (93.3)	0.319
SLEDAI	2 (0–4)	8 (5−10)	6 (4−16)	0.9
APS	52±29	38±32	14±23	0.185
dsDNA abs, UI/mL*	9 (5−26)	11 (5−31)	5 (5−15)	**0.002**
C3, g/L	1±0.3	1±0.3	1.1±0.3	**0.004**
HCQ	158 (88.3)	105 (89.0)	53 (86.9)	0.866
CS	101 (56.4)	64 (54.2)	37 (60.7)	0.508
CS current dosage, mg	5 (0–5)	5 (0–5)	5 (0–5)	0.145
MTX	23 (12.8)	105 (89.0)	50 (82.0)	0.182
AZA	5 (2.8)	4 (3.42)	1 (1.64)	0.662
MMF	22 (12.3)	14 (11.9)	8 (13.1)	0.999
CT imaging				
CAC score	51±188	17±81	117±291	**0.010**
CAC class				**0.006**
0	124 (69.3)	89 (75.4)	35 (57.4)	
1–100	39 (21.8)	24 (20.3)	15 (24.6)	
>100	16 (8.9)	5 (4.2)	11 (18)	
EAT volume	107±65	73±26	174±66	**<0.001**
Indexed EAT volume	63±37	43±14	101±39	**<0.001**
EAT density, HU	−79±4.4	−77±4	−82±4	**<0.001**

Categorical variables are expressed as n (%) and compared with χ2 tests or Fisher’s tests. Continuous variables are expressed as mean±SD deviation or median (IQR) and compared with Student’s t-test or Wilcoxon’s rank test. Bold results are statistically significant at p<0.05 level.

Prior ASCVD includes prior acute coronary syndrome, ischaemic stroke or transient ischaemic attack and symptomatic peripheral artery disease.

* dsDNA antibodies were obtained using Farr assays.

APS, antiphospholipid antibody syndrome; ASCVD, atherosclerotic cardiovascular diseases; AZA, azathioprine; CAC, coronary artery calcium; CS, corticosteroids; dsDNA abs, double-stranded DNA antibodies; EATi, indexed epicardial adipose tissue; HCQ, hydroxychloroquine; HDL-C, high-density lipoprotein-cholesterol; HU, Hounsfield units; IMT, intima-media thickness; inv., involvement; LDL-C, low density lipoprotein-cholesterol; MMF, mycophenolate mofetil; MTX, methotrexate; SLEDAI, Systemic Lupus Erythematosus Disease Activity Index; TC, total cholesterol; TG, triglycerides.

### EAT in patients with SLE

Mean EAT and EATi volumes were 107±65 mL and 63±37 mL/m^2^, respectively. EATi volume was increased (>68 mL/m^2^) in 61 patients (34.1%). Classification of patients according to EATi volume revealed that prior ASCVD rates, BMI, total cholesterol and TG levels were higher in patients with an increased EATi volume on univariate analysis (table 1). Interestingly, significantly elevated CRP concentrations, intima-media thickness, presence of carotid plaques and increased CAC score were seen in patients with an EATi volume >68 mL/m^2^. It is noteworthy that circulating dsDNA antibodies were lower and C3 levels were higher in the group of SLE patients with increased EATi volume. On multivariable analysis, age (β=0.91, p<0.001), BMI (β=1.1, p=0.02) and TG levels (β=18.4, p=0.009) were independently associated with EATi volume (table 2). Taken together, these findings indicated that a high EATi volume is frequently seen in patients with SLE where it is accompanied by an elevation of several key markers of cardiovascular diseases.

**Table 2:** Identification of independent clinical and biological predictors of index epicardial adipose tissue volume on multivariable analysis

	Univariate analysis		Multivariable analysis	
	β	P value	β	P value
Age at inclusion	1.6	<0.001	0.9	**<0.001**
Hypertension	30.1	<0.001	6.3	0.33
BMI	2.2	<0.001	1.1	**0.02**
TG	37.3	<0.001	18.4	**0.009**
HDL-C	11.4	0.48	9	0.54
LDL-C	31.4	<0.001	7.9	0.31
Diabetes	6.3	0.12	1.7	0.59
Carotid plaque	37.3	<0.001	13.7	0.08
C3 levels	40.4	<0.001	12.1	0.26
dsDNA abs	−0.24	0.01	−0.08	0.40
			**Model metrics**	
Multiple R^2^	–		0.50	
Adjusted R^2^	–		0.45	
P value	–		<0.001	

Bold results are statistically significant at p<0.05 level.

BMI, body mass index; dsDNA abs, double-stranded DNA antibodies; HDL-C, high density lipoprotein-cholesterol; LDL-C, low density lipoprotein-cholesterol; TG, triglycerides.

### Metabolomics and lipidomics signature in patients with elevated EAT

**Figure 1:**
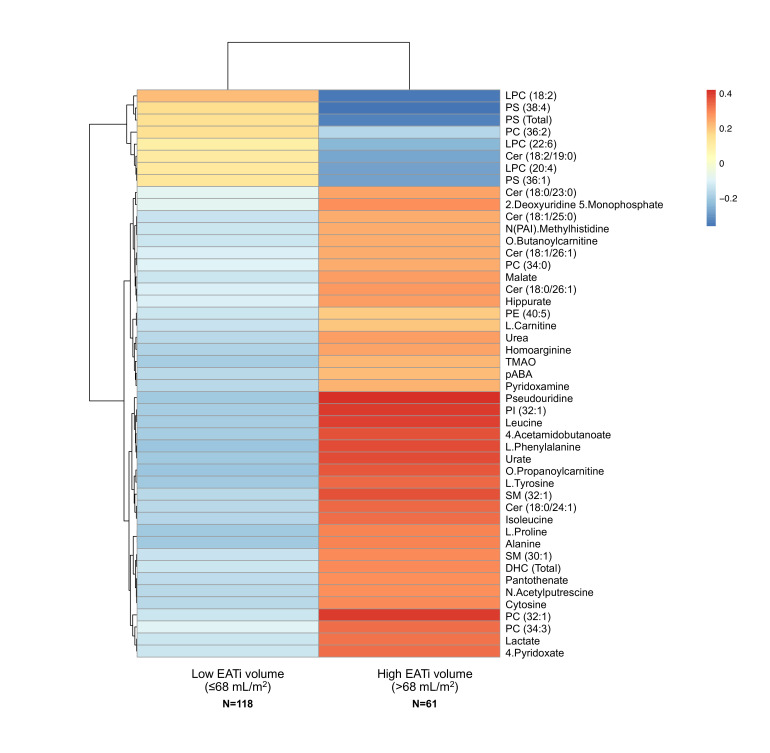
Heatmap of metabolomic and lipidomic features according to epicardial adipose tissue volume. All metabolomic and lipidomic features displayed are significantly (p<0.05) associated with epicardial adipose tissue volume status (ie, ≤ vs >68 mL/m^2^). Cer, ceramide; DHC, dihydroceramide; EATi, indexed epicardial adipose tissue; LPC, lyso-phosphatidylcholine; pABA, para-aminobenzoic acid; PC, phosphatidylcholine; PE, phosphatidylethanolamine; PI, phosphatidylinositol; PS, phosphatidylserine; SM, sphingomyelin; TMAO, trimethylamine N-oxide.

To determine whether a specific metabolomics and lipidomics signature was associated with EATi volume, amounts of circulating metabolites and lipid species quantified by LC-HRMS and LC-MS/MS, respectively, were analysed in patients with SLE according to the EATi volume threshold (≤ or >68 mL/m^2^). As a result, a total of 235 metabolites’ expression levels were compared between patients with increased EATi volume versus patients without. Heat map representation revealed that 47 of them (35%) were significantly associated with EATi status (all p<0.05) (figure 1). Of these, circulating levels of 39/47 metabolites and lipid species were significantly increased, including sphingolipids (ceramide (Cer), ceramide precursor dihydroceramides (DHC) and sphingomyelin species), metabolites related to amino acid metabolism, glycolysis, acylcarnitines and microbiota. Conversely, circulating levels of only eight lipid species were significantly decreased in this subgroup, mainly phosphatidylserines ((PS), total PS class, PS (38:4) and PS (36:1)) and lyso-phosphatidylcholines ((LPC), LPC (18:2), LPC (22:6) and LPC (20:4)).

**Figure 2:**
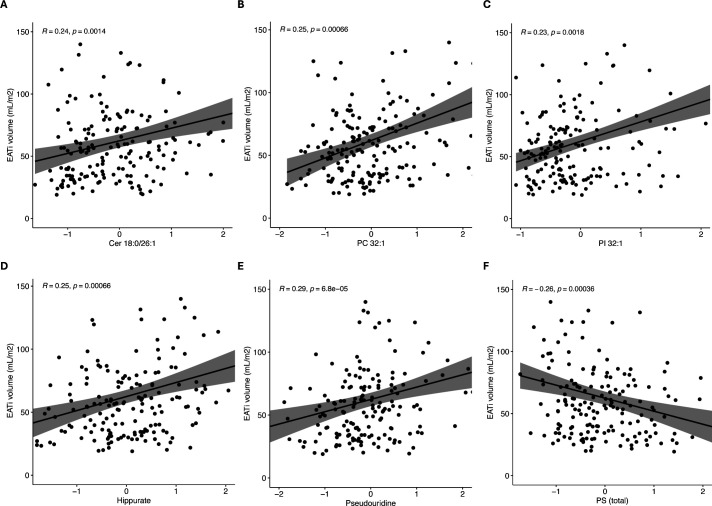
Scatter plots showing correlations between indexed epicardial adipose tissue (EATi) volume and key metabolites identified through Lasso regression. (**A**) Cer(d18:0/26:1), (**B**) PC(32:1), (**C**) PI(32:1), (**D**) Hippurate, (**E**) Pseudouridine and (**F**) Total PS class. Correlation coefficients (**R**) and Spearman correlation tests (p value) are reported. All correlations were positive except for total PS class. Cer, ceramide; PC, phosphatidylcholine; PI, phosphatidylinositol; PS, phosphatidylserine.

To further assess the relationship between circulating species and EATi volume irrespective of a predefined cut-off, a stepwise linear regression was performed (table 3). Overall, circulating levels of Cer (18:0; O2/26:1), phosphatidylinositol ((PI), PI (32:1)), total PS class, hippurate and pseudouridine were independent predictors of EATi volume in patients with SLE (table 3). Correlation plots are reported in figure 2 and show significant although weak correlations.

**Table 3:** Lipids and metabolites independently associated with indexed epicardial adipose tissue volume on stepwise linear regression analysis

	Lasso regression	Stepwise linear regression	
	Coefficients	β	P value
Cer (18:0/26:1)	1.6	7.9	**<0.001**
PC (32:1)	0.6	–	–
PI (32:1)	6.9	13.3	**<0.001**
Total PS class	−1.8	−8.5	**<0.001**
Hippurate	2.1	6.9	**0.005**
Pseudouridine	0.3	4.8	**0.048**
Other metabolites (n=229)	0	–	–
		**Model metrics**	
Multiple R^2^	–	0.37	
Adjusted R^2^	–	0.35	
P value	–	<0.001	

Bold results are statistically significant at p<0.05 level.

All 235 metabolites were included in the lasso regression. Variables with non-zero coefficients are shown.

Cer, ceramide; PC, phosphatidylcholine; PI, phosphatidylinositol; PS, phosphatidylserine.

Taken together, these data demonstrated that EATi volume was associated with an altered circulating lipidome and metabolome signature in SLE patients.

### Prediction of EAT in patients with SLE

**Figure 3:**
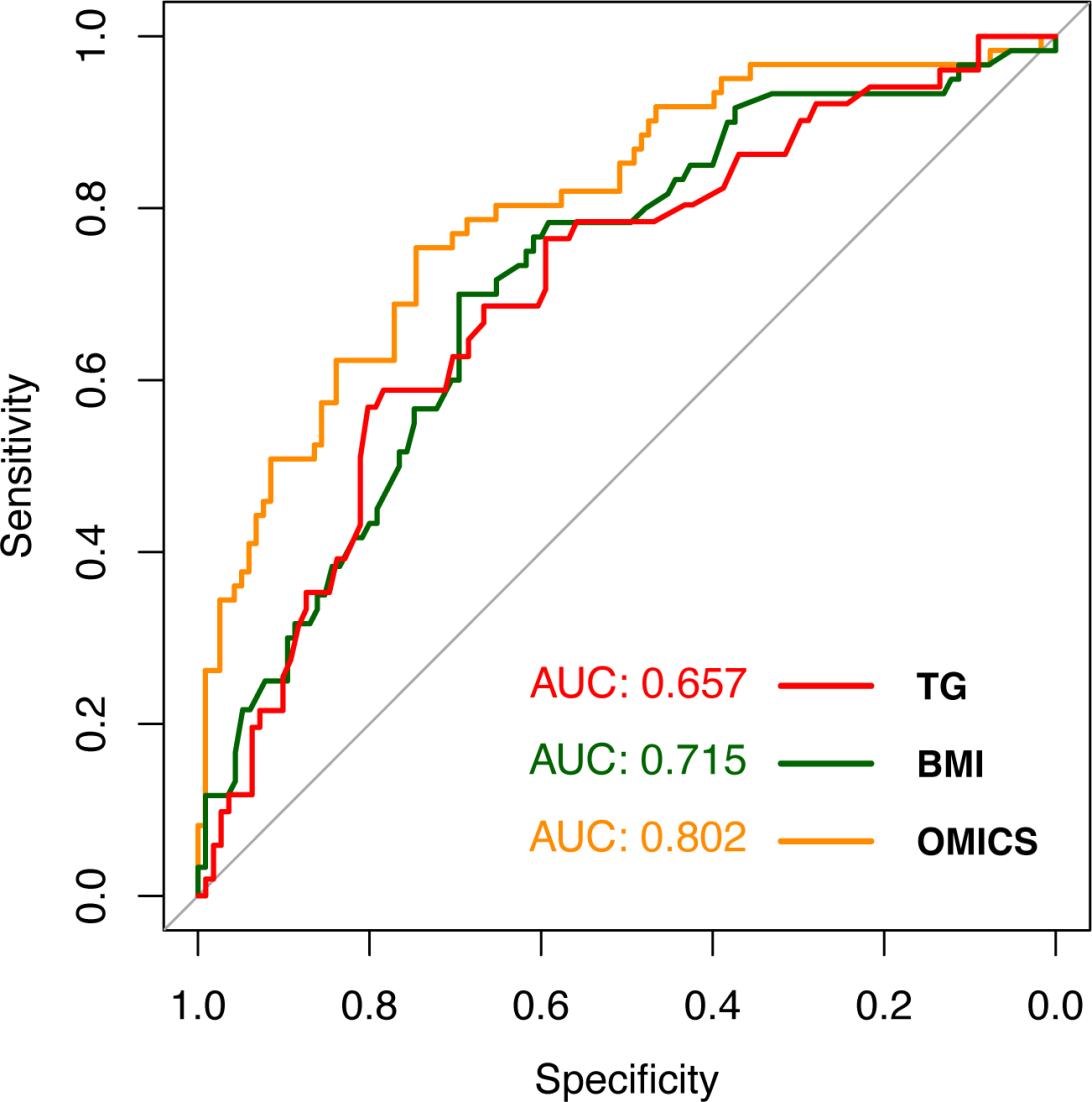
Receiver operating characteristic curve for EATi volume prediction (≤ vs > 68 mL/m^2^). AUC, area under the curve; BMI, body mass index; EATi, indexed epicardial adipose tissue; TG, triglycerides.

The performances in predicting EATi volume status (≤ vs >68 mL/m^2^) were compared for each candidate biomarker using area under the curve (AUC) ROC curve analyses. The AUC (95% CI)) and p value (when compared with omics) for EATi volume status prediction was 0.80 (0.73 to 0.87) for the omics signature (first dimension of the PCA), 0.72 (0.64 to 0.80) for BMI (p=0.07) and 0.66 (0.57 to 0.75) for TG levels (p=0.008) (figure 3). This analysis highlights the high capacity of lipidome and metabolome to predict EATi volume in SLE.

### Coronary calcium and EAT in patients with SLE

**Figure 4:**
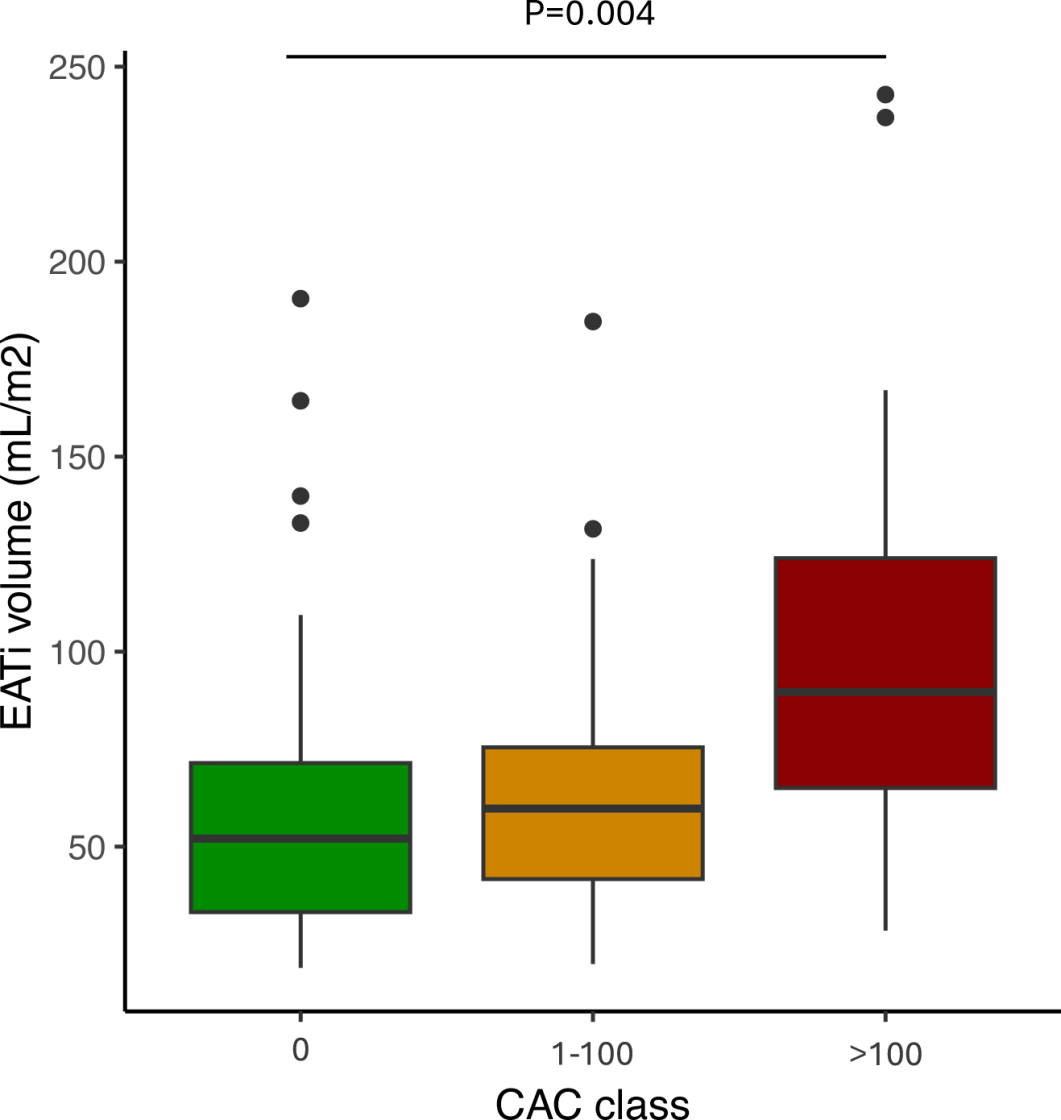
Association between EATi volume and CAC score. EATi volume according to CAC class (0, 1–100 and >100). CAC, coronary artery calcium; EATi, indexed epicardial adipose tissue.

Because EAT volume was reported to be associated with cardiovascular diseases in the general population, we next investigated if such an effect was observed in SLE. A modest correlation was detected between EATi volume and CAC score (ρ=0.32, p<0.001) in our SLE cohort. As shown in figure 4, EATi was significantly increased according to the CAC classes (low, CAC=0, moderate, 0< CAC <100 and high, CAC ≥100, p=0.004).

## Discussion

Our study demonstrated that EAT was associated with a specific circulating lipidomic and metabolomic signature in a cohort of 179 patients with SLE. Identification of lipid species and metabolites predicting EAT highlighted the importance of very-long chain DHC, PS and microbiota and suggested that these biomarkers could enhance cardiovascular risk stratification owing to their relationship with EAT volume.

Alteration of metabolic pathways is classically observed in SLE and contributes to associated disorders including cardiovascular diseases.^10^ In agreement with Lipson *et al*,^6^ we observed that BMI and TG levels were associated with EATi volume in patients with SLE. However, in contrast to this previous study, high-density lipoprotein-cholesterol levels were not significantly associated with EATi volume in our cohort of patients.

Hydrolysis of TG by lipoprotein lipase (LPL) leads to the release of free fatty acids which are used for energy supply, storage or lipid synthesis including sphingolipids.^22^ LPL activity was higher in EAT than in subcutaneous adipose tissue from patients with CAD.^23^ Interestingly, LPL activity was correlated with the ceramide content in EAT^23^; this latter being increased in EAT from patients with CAD.^9^ In our study, levels of circulating ceramide precursors—DHC—were positively associated with EAT volume in SLE. This suggests that, in SLE, ceramide accumulation in EAT is likely driven by uptake from circulation rather than local ceramide synthesis. Strikingly, DHC were strong predictors of CAC in our cohort of patients^10^ suggesting that DHC with very long-chain fatty acids could promote CAD through action in EAT. Indeed, ceramides are involved in several cellular pathways contributing to atherosclerosis, including inflammation,^24^ which could serve as an atherogenic belt between EAT and coronary arteries for promoting CAD in SLE. As a whole, our data pointed out the importance of ceramide precursors, DHC with very long-chain fatty acids, in the association between EAT and CAC and the prediction of CAD in SLE.

This communication between EAT and coronary atherosclerosis is also supported by other lipid species (PS class and PI (32:1)) associated with both EAT (present study) and CAC (in our previously published cohort of patients with SLE^10^). Although the implication of PI (32:1) in this effect remains to be elucidated, these findings reinforce the protective action of PS in numerous pathways involved in atherosclerosis and CAD.^25^ Finally, in agreement with the intestinal dysbiosis described in patients with SLE,^26^ our study shows that concentrations of several microbial metabolites were associated with the volume of EAT. Among them, hippurate, a product of microbial carbohydrate fermentation whose circulating levels are altered in SLE in comparison to healthy individuals,^27^ was a strong independent predictor of the EATi volume in SLE. Strikingly, hippurate was equally positively associated with the degree and the presence of CAC in this cohort of patients with SLE.^10^ A similar observation can be made with pseudouridine, a modified nucleoside involved in numerous biological functions,^28^ whose elevated plasma concentrations were observed in patients with CVD.^29 30^ Overall, the identified correlations between EATi volume and lipid species remained weak although significant.

Most patients with a CAC score above 100 exhibited high EATi volume, whereas EATi volume showed a more heterogeneous distribution among patients with CAC scores below 100. This variability likely explains the relatively weak overall correlation between the two measures and suggests that EATi volume may help refine cardiovascular risk stratification in patients with low CAC scores by identifying individuals at higher risk despite limited coronary calcification. In a previous study involving a cohort of SLE patients, we demonstrated that EATi volume provided incremental prognostic value over CAC score.^31^ Such prognostic value may be related to the fact that EATi volume is associated with both the presence and the extent of non-calcified coronary plaques.^32 33^ These findings may also suggest that some patients with high CAC but low EATi develop atherosclerosis through alternative risk pathways.^10^

As a whole, our study highlights the strong interaction between the circulating lipidome/metabolome and the EAT and identified shared predictors of both EAT and CAC in SLE. However, further studies are needed to validate that this circulating signature is associated with a modification of lipid and metabolic pathways in EAT.

Cardiovascular stratification of SLE patients remains a challenging unmet need.^1 3 4^ Previous data have shown that most risk prediction tools underestimated the real cardiovascular risk of these patients.^4 14 34^ Clinical risk scores underperform in SLE and may lead to missing high-risk patients.^14^ Further, patients with SLE frequently display non-calcified coronary plaques.^35 36^ To that extent, assessing CT-derived EATi volume could enhance the performance of non-contrast cardiac CT by upclassifying zero CAC patients that are yet at high risk.^5^ Owing to the relationship between EATi volume and atherosclerosis, EATi volume could be used to enhance risk stratification in SLE patients. Overall, CT-derived EATi volume could be used to enhance risk stratification in SLE patients starting after 40–50 years old.^5 31^ An opportunistic approach leveraging CT scans performed for other indications could further reduce radiation exposure in this population.^37^

From a preventive clinical standpoint, these hypotheses, if confirmed, could warrant the assessment of EAT volume to guide the medical management of cardiovascular risk in SLE patients at early stages of the disease. Currently available pharmacological approaches targeting EAT include sodium-glucose cotransporter 2 inhibitors (SGLT2i), glucagon-like peptide-1 receptor agonists (GLP-1 RA) and lipid-lowering therapies.^7^ The use of such molecules to mitigate cardiovascular risk deserves further studies in SLE.

This study has limits. It was a retrospective single-centre study with a potential selection bias. All patients were female. No follow-up data were reported. No association between future incident clinical events and biomarkers (omics or EATi volume) was reported. Direct comparisons between omics, EAT volume and existing cardiovascular risk stratification tools, including risk scores, lipoprotein(a), ultrasound-based biomarkers such as resistance and pulsatility indices^38 39^ or intima-media thickness, were not possible. This study has strengths. Sample size was large. All patients benefitted from both CT and omics assessment.

## Conclusion

This study identified key cellular pathways and biomarkers associated with increased EAT volume in SLE. Assessing and targeting cardiometabolic risk factors could help mitigate cardiovascular risk in SLE patients but warrants further research.

## Supplementary material

10.1136/lupus-2026-001993 online supplemental file 1

## Data Availability

Data are available upon reasonable request. Data may be obtained from a third party and are not publicly available.
