# Supplementary material for: Serum lipidomic and metabolomic signatures link epicardial adipose tissue to cardiovascular diseases in SLE: a post-hoc analysis
Source: Lupus Sci Med. 2026 Apr 21;13(1):e001993. doi: 10.1136/lupus-2026-001993 (PMC13141169; doi:10.1136/lupus-2026-001993)
Supplement: online supplemental file 1 [file lupus-13-1-s001.pdf]

Metabolomic signatures associated with epicardial adipose tissue in systemic lupus erythematosus

Study design

French SLE Referral Center

Monocentric
Retrospective
Cross-sectional

Inclusion criteria

SLE with cardiac CT and metabo/lipidomics assessment

EATi volume

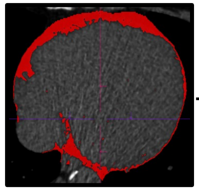

>68mL/m²

≤68mL/m²

Metabo/lipidomics

Main results

Study population

N=179 SLE patients

Female sex 100%

Age 43±14 years-old

CAC score 51±188

EAT volume 107±65 ml

EATi vol. >68mL/m², 34%

Key metabolomic and lipidomic pathways associated with increased EATi volume

Overexpression	Underexpression
Very long-chains ceramides	Phosphatodylserines
Aminoacid metabolits	Lysophosphatidylcholine
Fatty acids oxydation	
Microbiota-related metabolites	
Nonoxydative glycolysis	
